# Clinical charts for surveillance of growth and body proportion development in achondroplasia and examples of their use

**DOI:** 10.1002/ajmg.a.61974

**Published:** 2020-11-21

**Authors:** Luitgard Neumeyer, Andrea Merker, Lars Hagenäs

**Affiliations:** ^1^ Pediatric Endocrinology Unit, Karolinska University Hospital Stockholm Sweden; ^2^ Department of Women's and Children's Health Karolinska Institutet Stockholm Sweden

**Keywords:** achondroplasia, clinical surveillance, growth chart

## Abstract

Clinical surveillance of infants and children with achondroplasia necessitates syndrome‐specific charts due to extreme short stature with deviating body proportions. Height, arm span and leg length develop far below normal population ranges. We present growth and body proportion charts for ages 0–20 years, constructed from semi‐longitudinal standardized measurements of about 450 children, along with some examples of achondroplasia typical and atypical growth pattern. We combine head circumference, height and weight for 0–4 years into one (infancy) page and height and weight for 4–20 years in another (childhood–adolescence) using nonlinear axes to account for the rapidly decreasing growth velocity. Similarly, weight and BMI are based on nonlinear axes to balance wide SD‐channels at higher and narrow SD‐channels at lower levels of weight/BMI. Charts for following sitting height, sitting height/height ratio, arm span, leg and foot length are also presented. Clinical examples illustrating the applicability of the charts include cases of extreme prematurity, extreme head circumference development before and after shunting, achondroplasia complicated by chromosomal or additional genetic abnormality and by growth hormone deficiency as well as of evaluating growth promoting therapy.

## 1 INTRODUCTION

For achondroplasia the use of syndrome‐specific growth charts are necessary since height develops far below normal curve area and body proportions are profoundly distorted due to the extreme shortness of the extremities in comparison with a nearly normal trunk. Despite almost normal birth length and growth development during first postnatal months, length position in the normal population chart decreases fast to about −5 standard deviation scores (SDS) after the first year of life. This position is mainly maintained during childhood but decreases further over puberty to about −6 SDS in adulthood. This implies that a 7 year old boy or girl with achondroplasia is on average about 26 cm shorter than normal population (WHO reference [de Onis et al., 2007]) and as adults 44/39 cm male/female (M/F) shorter (Merker, Neumeyer, Hertel, Grigelioniene, Mäkitie, et al., 2018).

Head size is most often greatly increased and shows accelerated growth in early infancy; birth size averages about +1.5 SDS accelerating to +2 SDS at 1 year and + 3 SDS at 2 years of age (British reference [Cole, Freeman, & Preece, 1998]). Brain tissue volume is at average clearly increased in achondroplasia (Thompson et al., 1999) but also ventricular and extra‐cerebral liquor spaces are commonly slightly increased giving an impression of communicating hydrocephalus. A pronounced acceleration of head size merits investigation for possible liquor circulation problems necessitating surveillance for hydrocephalic development.

Deviant body proportions in achondroplasia can be observed already during infancy; at 2 years of age are arm span and leg length more than 10 cm shorter than in normal infants. Normal population arm span, that initially is short relative to height, roughly equals height from early school ages up to adulthood (Engelbach, 1932; Gerver & de Bruin, 2011). In achondroplasia, however, this initial arm span “deficit” relative to height instead increases with age, from about −8 cm at 7 years of age to −10/−14 cm in M/F adults (Merker, Neumeyer, Hertel, Grigelioniene, Mohnike, et al., 2018). Mean adult arm span in achondroplasia is 122/110 cm M/F compared to 186/174 cm in normal population (Gerver & de Bruin, 2011). Leg length in achondroplasia is at 7 years of age 28 cm shorter than in normal population (Fredriks et al., 2005) and 44/40 cm M/F in adulthood. Deviation in sitting height is usually only minor, 3 cm less at 7 years and 7/6 cm M/F at adult ages, and thus contribute to an extreme sitting height/height ratio of 66/67%, which is above +10 SDS, (Merker, Neumeyer, Hertel, Grigelioniene, Mohnike, et al., 2018) compared to 51/53% in the normal population (Fredriks, van Buuren, van Heel, et al., 2005). Due to the combination of extreme short legs and a near normal trunk length, body mass index (BMI) value becomes greatly distorted. Therefore, the perception of excess weight and thus risk for overweight and obesity might be increased in achondroplasia.

Achondroplasia‐specific growth references are available from Europe, USA, Japan, Argentina and Australia, yet their presentations might be of reduced value in clinical practice due to small chart size and poor resolution. Non available tabled values (Hoover‐Fong et al., 2007, 2008; Horton, Rotter, Rimoin, Scott, & Hall, 1978; Hunter, Hecht, & Scott, 1996; Hunter, Reid, Pauli, & Scott, 1996; Nehme, Riseborough, & Tredwell, 1976; Tofts, Das, Collins, & Burton, 2017) or limitation to certain age groups or anthropometric variables (Hoover‐Fong et al., 2017; Tachibana, Suwa, Nishiyama, & Matsuda, 1997), restrict reproducing some references into clinical growth charts. We therefore constructed clinical growth charts from our European achondroplasia references (Merker, Neumeyer, Hertel, Grigelioniene, Mäkitie, et al., 2018; Merker, Neumeyer, Hertel, Grigelioniene, Mohnike, et al., 2018) to facilitate their implementation into clinical practice. The aim here is to present these clinical charts and to show their use by sharing examples from clinical practice.

## 2 MATERIALS AND METHODS

From the material described in Merker, Neumeyer, Hertel, Grigelioniene, Mäkitie, et al. (2018) and Merker, Neumeyer, Hertel, Grigelioniene, Mohnike, et al. (2018), clinical charts were constructed covering ±3 *SD* for measured (length/height, weight, head circumference, sitting height, arm span, foot length) and derived (leg length and BMI) anthropometric variables. These references were retrieved based on 4.375 measuring occasions on length/height, weight and head circumference (466 individuals) and 1.705 for body proportion (382 individuals). The great majority of measurements were conducted by the same observer (LN) using standardized technique during routine clinical visits at the skeletal dysplasia clinic at the Department of Pediatrics at Karolinska University Hospital in Stockholm, Sweden and at annual meetings of German Association for People of Short Stature and their Families. Sex‐specific growth references were modeled with GAMLSS package in R Version 3.2.3 (Rigby & Stasinopoulos, 2005) using the LMS‐method, which summarizes the distribution of each anthropometric variable for age by three curves: Box‐Cox power transformation (L), the median (M) and the coefficient of variation (S) (Cole & Green, 1992). Final model choice was based on model fit, evaluated by QQ‐plot, as well as curve appearance.

The clinical growth charts were constructed in cooperation with PC PAL, a company specialized in growth module software. Achondroplasia typical and atypical growth pattern were chosen to show the usability of these charts. The cases had been followed by the skeletal dysplasia clinic at the Department of Pediatrics at Karolinska University Hospital in Stockholm, Sweden. Standard deviation scores (SDS/*z*‐scores) were calculated to evaluate changes in the achondroplasia chart and in some cases also to describe deviations from the normal population (WHO reference [de Onis et al., 2007; WHO Multicenter Growth Reference Study Group, 2006]).

This study was approved by the ethical committee at Karolinska Institutet.

## 3 RESULTS

We choose to construct ±3 *SD* charts covering ages 0–20 years for length/height, weight, head circumference and BMI and 2–20 years for body proportion variables (3–20 years for foot length). These are prepared to be printed as A3 format, with two pages per sheet arranged, that is folded to an A4 booklet. These growth chart booklets can be accessed from Supporting information S1 and S2.

### 3.1 Introducing the charts

Adapted from the layout of the Swedish growth charts (Engstrom et al., 1973) we combine charts for head circumference, length/height and weight for ages 0–48 months on one (infancy) page as well as height and weight for ages 4–20 years also on one (childhood‐adolescent) page. This is achieved by using non‐linear age y‐axes that also increase plotting and reading sensitivity while keeping the number of pages at a minimum. The idea of the chart layout is to combine both 0–4 and 4–20 years into one view, as exemplified in subsequent clinical examples, since the entire growth pattern can give valuable information. Normal population mean for 0–5 years (WHO Multicenter Growth Reference Study Group, 2006) and 5–19 years (de Onis et al., 2007) is shown in both chart pages for orientation. A head circumference chart for ages 0–20 years is added in a separate page to the chart compilation enabling longer individual follow‐up. This chart also uses nonlinear axes to give attention to head circumference development during the first years of age.

Similarly, we manipulate the BMI‐axis with a logarithm that gives a balanced attention to upper and lower BMI ranges. On linear scales more chart space is dedicated to the higher BMI ranges that, due to the right skew in the BMI distribution, are broader than at lower BMI. Yet, BMI development at low levels might also require clinical attention, which is why we adapt this approach from the layout of the Swedish BMI reference (Karlberg, Luo, & Albertsson‐Wikland, 2001) charts. International Obesity Task Force (IOTF) cut‐offs for overweight and obesity (Cole & Lobstein, 2012) are added for orientation, which overlap with normal achondroplasia BMI ranges.

An overview of body proportions comprises both absolute and relative sitting height charts as well as arm span and leg length charts. These cover ages 2–20 years on linear axes. Normal population mean (Fredriks, van Buuren, van Heel, et al., 2005) are shown for orientation in both sitting height charts; for relative sitting height are also +2 *SD* indicated delimiting normal body proportion range.

### 3.2 Clinical examples

To show the applicability of these charts and to support their clinical implementation, we present some examples of achondroplasia typical and atypical growth pattern. Note that selected patient examples are only to show the use of our clinical charts and may not be representative for respective condition.

#### 3.2.1 Healthy growth pattern

**FIGURE 1:**
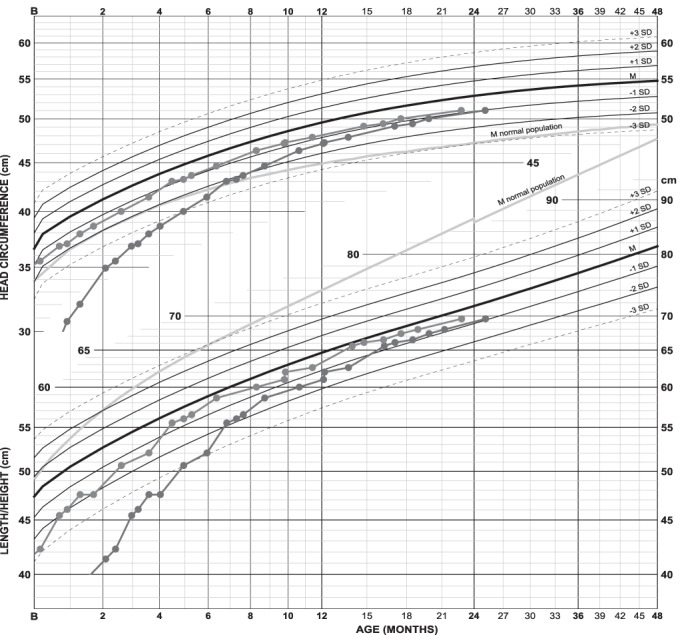
Growth chart of a girl with achondroplasia and extreme prematurity: Length and head circumference are shown both uncorrected (in red) and corrected (in blue) for 10 weeks prematurity. A catch‐up in length is seen during the first 4 months. The difference between both curves naturally diminishes with age since degree of adjustment shrinks on the age scale. Length is at 0, 6 months, and 2 years of age respectively −2.7, −0.3, and −0.4 SDS. Without age correction length would be −5.9, −3.0, and −0.9 SDS [Color figure can be viewed at wileyonlinelibrary.com]

Length and head circumference development of a girl with achondroplasia and extreme prematurity (Figure 1) highlights the result of adjusting for gestational age. With birth length of 35 cm and head circumference of 26 cm, she clearly deviates from term typical achondroplasia newborns (in red) but seems to catch up fast. When gestational age (29 weeks +6 days) is taken into account (in blue), however, her growth pattern appears quite normal. She has, for achondroplasia, an early motor and cognitive development, walking independently at 17 months uncorrected age (motor development scheme not shown).

#### 3.2.2 Aberrant growth pattern

**FIGURE 2:**
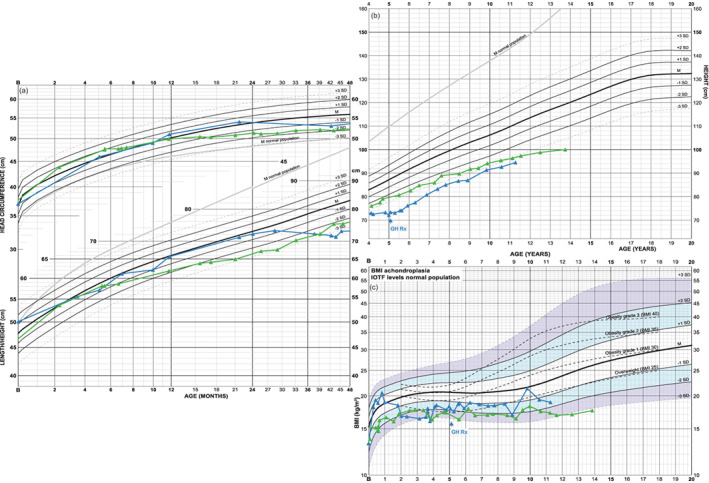
Growth charts of two boys with atypical achondroplasia height pattern: Growth of the boy in green is probably influenced by foramen magnum stenosis with atrophy of medulla oblongata with paraplegia and hypoventilation requiring nighttime respiratory support. His length develops at the lower extremes of achondroplasia, that is, at −3 SDS as of 2 years of age, mainly due to limitations of leg growth (shown in Supporting information S3). His head grows normally until 12 months of age but slows down thereafter resulting in a “loss” in growth chart position; head circumference SDS at 12, 16, and 43 months respectively: +0.1, −0.6, −2.0 SDS. Note that his curve is corrected for 6 weeks prematurity. Height development in the other boy (in blue), probably growth hormone (GH) deficient and also with failure to thrive, stops at about 2 years of age resulting in a change of height position from −0.4 SDS to −4.5 SDS at 5 years of age, when GH therapy (GH Rx) is initiated. Following treatment start, a partial catch‐up growth is seen, −3.7 SDS at 7 years of age, mainly depending on increased position in sitting height (shown in Supporting information S3) [Color figure can be viewed at wileyonlinelibrary.com]

Two examples show how growth can deviate and develop outside normal range (i.e., ±2 *SD*) necessitating closer clinical surveillance (Figure 2). Timing of deceleration as well as underlying cause differs between the cases. One of the boys (in green) is paraplegic due to foramen magnum stenosis causing myleomalacia also needing night time respiratory support due to hypoventilation. The other boy (in blue) is a case with probable growth hormone (GH) deficiency, possibly also connected to failure to thrive. Linear growth is nearly absent from 2 years of age until start of GH treatment at 5 years. BMI in both boys develops clearly in lower achondroplasia ranges; −3.2 SDS at 13.9 years (in green) and −1.6 SDS at 11.3 years (in blue) of age.

#### 3.2.3 Achondroplasia including syndromic conditions

**FIGURE 3:**
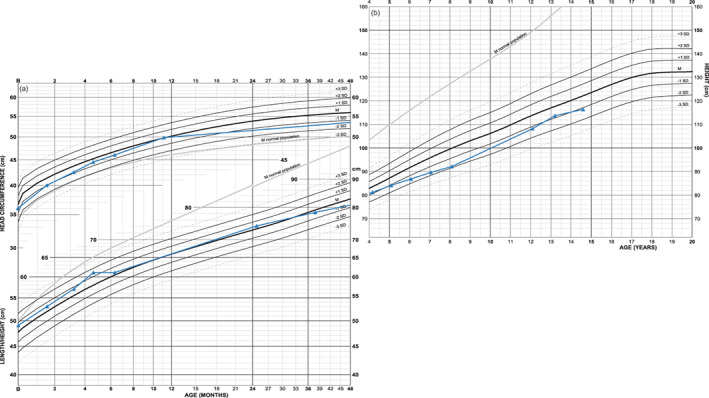
Growth chart of a boy with achondroplasia and Mb Down: Length follows strictly achondroplasia mean during the first postnatal years. Thereafter height position decreases to −2 SDS but it is unclear whether this decrease is dependent on the trisomy or on a temporary slower tempo of growth during childhood. Body disproportion develops in a milder direction than is typical for achondroplasia (charts found in Supporting information S4) [Color figure can be viewed at wileyonlinelibrary.com]

**FIGURE 4:**
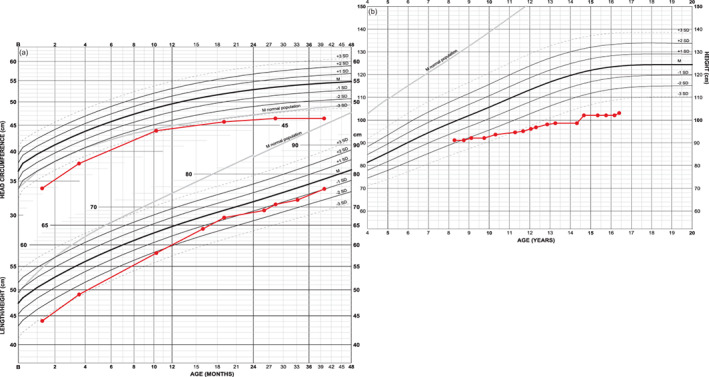
Growth chart of a girl with achondroplasia and Kabuki syndrome: She is small at birth and seems to catch‐up from outside achondroplasia normal ranges to −1 SDS at about 18 months of age. Note, however, that length position in achondroplasia typically rapidly falls during the first year of life and that her postnatal length curve develops in parallel to normal population mean (gray orientation line in the chart) indicating that intrauterine growth might be affected and that the same length position is retained during infancy and childhood; after 1 month, at 12 months and after 18 months respectively −3.2, −2.0, and −1.1 SDS in the achondroplasia chart versus −5.5 SDS at all three age points in the WHO reference. Final height seems to be reached at 15 years of age; height is 102 cm at 14.7 years of age or −4.8 SDS in the achondroplasia chart (−9.3 SDS in WHO reference) [Color figure can be viewed at wileyonlinelibrary.com]

Possible additive effect from presence of FGFR3 mutation and an additional syndromic condition is shown in a boy with achondroplasia and Down syndrome (Figure 3) and a combination of achondroplasia and Kabuki syndrome in a girl (Figure 4). In the first example is height not clearly affected; height deficits in Mb Down and achondroplasia seem not additive, at least not during infancy and early childhood. In the second example, birth size and pubertal growth are clearly subnormal while final height seems to be reached earlier than normal. Head circumference is also untypical for achondroplasia indicating that an additional syndromic condition might be present.

#### 3.2.4 Head circumference and the effect of shunting

**FIGURE 5:**
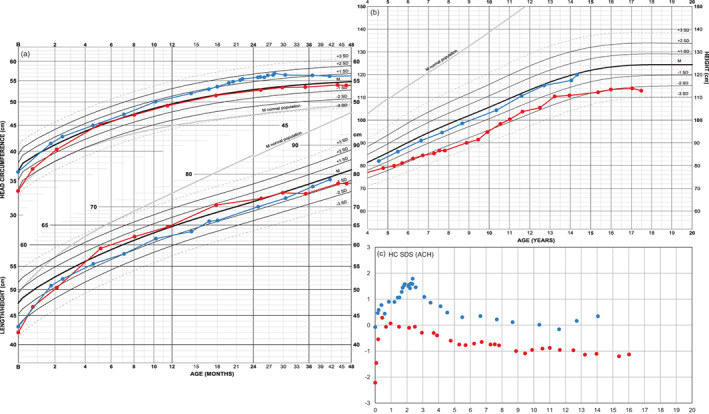
Growth charts and head circumference SDS curves of two girls both shunted at 2 years of age: The head grows slower in both girls after shunting resulting in a decrease in head circumference SDS. The increased tempi in head circumference growth could hypothetically influence tempo in height. Height in one girl (in blue) is −0.7 SDS at 2 years of age and improves to −0.4 SDS during childhood, approaching achondroplasia population mean during pubertal ages. Height position in the other girl (in red) falls from 0.1 SDS at 2 years of age to below normal ranges despite a temporary improvement caused by pubertal spurt. Adult height (114 cm) approaches prepubertal position at −2.1 SDS (−7.3 SDS in WHO reference) [Color figure can be viewed at wileyonlinelibrary.com]

Head circumference development of two girls, both with shunt from 2 years of age with different medical background (Figure 5). One girl (in blue) is shunted mainly due to accelerating head circumference; subsequently she also develops myelomalacia at craniocervical junction causing surgical intervention of foramen magnum. The other girl (in red) is shunted because of hydrocephalus. She also develops myelomalacia and atrophy at foramen magnum level necessitating surgical decompression. She is partially paraplegic and is dependent on night time respiratory support.

#### 3.2.5 Weight and BMI

**FIGURE 6:**
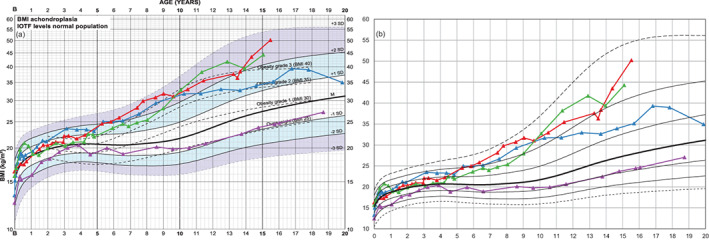
BMI charts of four boys with achondroplasia: (a) the nonlinear BMI‐axis causes upper BMI “channels” to be increasingly compressed compared to a linear axis (b). Three boys (in blue, green, red) with pronounced ability to gain weight and one boy (in purple) with more healthy weight and thus BMI development are shown. The boy marked in red gains 13 kg within 14 months, 23 kg within 22 months, weight increasing from +2.1 to +2.8 SDS (not shown), which pushes BMI from +2.0 to +2.7 SDS at 15.5 years of age. During the same period, the boy marked in purple gains 9 kg, changing from −0.8 to −0.4 SDS in the weight chart (not shown) or −1.0 to −0.9 SDS in the BMI chart [Color figure can be viewed at wileyonlinelibrary.com]

BMI development of three boys is shown (Figure 6) due to their ability to gain weight during a relatively short time. As comparison, an example of more healthy BMI development is added (in purple).

#### 3.2.6 Sitting height might be important for diagnosing

**FIGURE 7:**
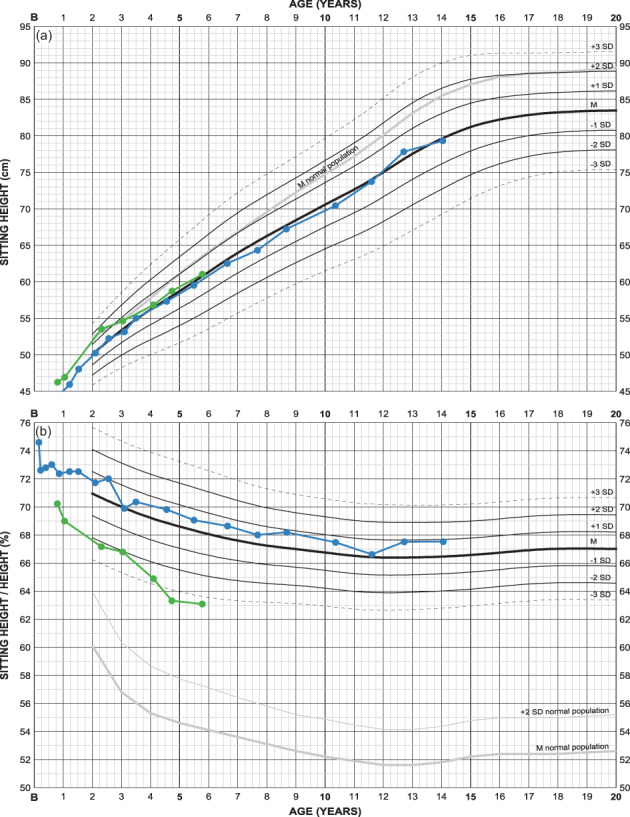
Sitting height charts of two girls with different FGFR3 mutations: one (in blue, from Figure 5) with Gly380Arg/achondroplasia and one (in green) with Asn540Lys/hypochondroplasia. At 6 years of age, sitting height is −0.1 and +0.1 SDS respectively at a relative sitting height of +0.5 and −3.4 SDS. Height corresponds to −0.3 and +1.9 SDS (shown in Supporting information S5) [Color figure can be viewed at wileyonlinelibrary.com]

Two examples of FGFR3 mutations illustrate the use of these charts (Figure 7); a girl with achondroplasia (in blue) and one with hypochondroplasia (in green). Absolute sitting height is almost identical in both girls while relative sitting height is significantly lower in the girl with hypochondroplasia. Her body disproportion becomes thus less severe.

#### 3.2.7 Arm span and (subischial) leg length should also be followed systematically

**FIGURE 8:**
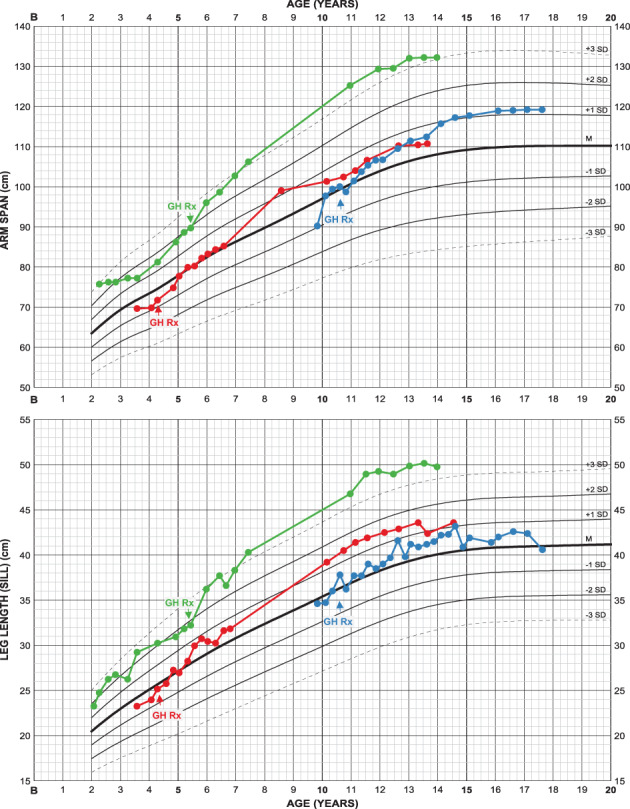
Arm span and leg length charts of three girls with achondroplasia, all treated with growth hormone (GH Rx) from indicated ages: Both arm span and leg length seem to be stimulated by the treatment ending up in favorable measures in all girls. Note that these cases are selected only to show the usability of the charts and cannot be regarded as representative for growth hormone treatment in achondroplasia. Corresponding height and sitting height charts can be found in Supporting information S6 [Color figure can be viewed at wileyonlinelibrary.com]

Growth of three girls treated with GH are illustrated (Figure 8) for which a positive treatment effect is observed on both arm span and leg length. It is unclear to what extent a better ability to stretch arms and legs might contribute to the measurements.

### 3.3 Other information to be documented in the charts

The chart collection also comprises check lists of important medical areas for prospective surveillance adapted from guidelines for health supervision in achondroplasia (Pauli, 2019; Trotter & Hall, 2005; Wright & Irving, 2012). In addition, we include a motor development scheme for achondroplasia (Todorov, Scott, Warren, & Leeper, 1981) to the infant page as well as pubertal stage scheme of normal population (Juul et al., 2006) to the childhood‐adolescent page since both can support evaluation of growth. Recommendations for measuring technique and important areas of medical surveillance are summarized in Supporting information S7.

Since BMI definitions of risk for overweight and obesity in achondroplasia are unclear, we added a waist circumference chart to the BMI page. To account for metabolic disease risk, waist circumference, as a measure of abdominal‐visceral fat, might be important to follow although reference data for achondroplasia is not available. Since trunk height is nearly normal in achondroplasia, it may be feasible to use normal population waist circumference data. A chart using reference data from 1997 Dutch Nation‐wide normal population survey also comprises hypothetical limits for overweight and obesity (Fredriks, van Buuren, Fekkes, Verloove‐Vanhorick, & Wit, 2005).

## 4 DISCUSSION

We present achondroplasia‐specific clinical charts in a layout intended to support clinical evaluation and surveillance. These charts reflect our experience from decades of clinical handling of this patient group.

### 4.1 Layout of growth charts to increase visual reading and plotting sensitivity

To compensate for the higher growth velocity at younger ages (Engstrom et al., 1973), we used a nonlinear axis for the ages 0–48 months making the curve area less compressed. In this way equal importance/resolution is given to the varying developmental velocities. It makes it possible to follow the individual growth pattern in great detail also during infancy when height velocity generally is highest. The use of nonlinear axes for some measured variables (*y*‐axes) adapts the curve area to the magnitude of the variable conferring sufficient visual and plotting sensitivity to allow for short time detection of abnormal growth pattern.

### 4.2 Anthropometric variables should be evaluated together

The use of nonlinear axes makes it also possible to combine several anthropometric variables in the same chart. The development of length, weight and head circumference can then be evaluated in comparison to each other; for example, length development in situations with extensive weight gain/loss or possible influence of significant head circumference acceleration. With the same intention we combined the clinical charts for the entire growth period in a two‐page format since the growth pattern during first years of life can retrospectively be informative when following the child at older ages.

### 4.3 Standard deviation versus centile

SDS (standard deviation score/*z*‐score) is clinically more useful and mathematically more manageable than centiles. The present charts cover an achondroplasia range from −3 to +3 *SD* corresponding to 0.135th to 99.865th centiles. Compared to background population (WHO reference [de Onis et al., 2007; WHO Multicenter Growth Reference Study Group, 2006]) children with achondroplasia typically follow a height position of −5 SDS during prepubertal ages attaining final adult height at about −6 SDS. In centiles this corresponds to 3.002 × 10^−5^ and 1.248 × 10^−7^. These SDS positions have no inherent biological meaning in comparison to normal population distribution but are a way of illustrating positions in an artificial background matrix. It is then possible to describe changes in positions with age; e.g., from −4.8 to −5.5 SDS which could be more informative or easier to handle than 11 cm below the third centile changing to 17 cm below third centile.

### 4.4 Motor development scheme and pubertal stage indicator

The chart booklet includes an achondroplasia motor development scheme adapted from Todorov et al (Todorov et al., 1981). Later publications (Fowler, Glinski, Reiser, Horton, & Pauli, 1997; Ireland et al., 2010, 2012) largely confirm those average ages for motor milestones and variability also seems to be similar. The 90th centile for attaining a function in achondroplasia may in fact sometimes represent individuals with neurological affection caused by a narrow foramen magnum or spinal canal. Illustrations of special techniques developed by the child with achondroplasia for locomotion is found in Fowler et al. (Fowler et al., 1997) and by the Sydney children's hospital network (The Sydney children's hospital network, 2016). Modern attitudes aim at postponing unsupported sitting and walking in order to minimize the risk for persistent spinal deformities until truncal stability is attained (The Sydney children's hospital network, 2016), which may, however, increase mean developmental ages somewhat.

There are no references for pubertal stage development in achondroplasia but clinical experience and few reports speak for normal pubertal timing (del Pino, Fano, & Adamo, 2018; Horton et al., 1978). A pubertal stage scheme in the chart enables to indicate age at attaining respective Tanner stage. This scheme shows mean and normal variation (±2 *SD*) for start of pubertal development and for menarche and is based on a recent Northern European reference (Juul et al., 2006).

### 4.5 Comparison to normal population with ethnic differences in height

Adult height seem to develop rather similar in different achondroplasia cohorts, suggesting that the FGFR3 mutation overrides natural ethnic variations (Merker, Neumeyer, Hertel, Grigelioniene, Mäkitie, et al., 2018; Tofts et al., 2017). Mean height in normal Dutch and Scandinavian population is for instance one *SD* higher than the WHO reference while Argentinian mean is one *SD* lower. These differences and limited availability of body proportion references might need to be taken into account when describing growth deviations in achondroplasia. UK references for sitting height and leg length (Dangour, Schilg, Hulse, & Cole, 2002) might be appropriate body proportion complement to growth references from the WHO since both reflect population of similar height. Yet, arm span references per se are rare, especially for the entire growth period from birth to adult ages, and references for relative sitting height are limited to studies from the Netherlands (Fredriks, van Buuren, van Heel, et al., 2005; Gerver & de Bruin, 2011) and Argentina (del Pino, Orden, Arenas, & Fano, 2017) which are indispensable for assessment of body disproportion and diagnosing of skeletal dysplasia. Thus, considering that the majority of differences in height is associated to leg length (Bogin & Varela‐Silva, 2010) it might appear somewhat unfair to compare leg length and arm span in achondroplasia to a very tall reference.

### 4.6 Achondroplasia and chromosomal abnormalities

We present a case with achondroplasia and Down syndrome, a combination which has been reported before (de Azevedo Moreira et al., 2010; Santos, Silva, & Pinto, 2011). In our example the growth modulating effect from an extra chromosome 21 seem to preferentially affect sitting height while leg length is average for achondroplasia. The combination achondroplasia‐Klinefelter syndrome is reported only in a few cases in literature, mostly without auxological data. It seems however that any growth stimulating effect from the extra X chromosome cannot override the limiting signaling from the mutated FGFR3 (Arditi et al., 2017).

### 4.7 A growth chart as a grid

Lastly, it is worthwhile noting that a growth chart only shows the distribution of a measured variable per age, as is the case in all growth references, and that any growth chart should only be seen as a background model or grid when following the individual. No child is necessarily expected to follow a specific centile line or “channel” and centile shifting is a normal and common phenomenon (Hermanussen, Largo, & Molinari, 2001; Mei, Grummer‐Strawn, Thompson, & Dietz, 2004) due to inherent differences in tempo of growth. Also on syndrome‐specific growth charts, an individual may follow his/her own growth pattern dictated by deviant maturational tempo. This would also be valid for achondroplasia (del Pino, Fano, & Adamo, 2019).

When height position is far below the normal population curve area and syndrome‐specific growth charts are not available, the use of an extreme “short stature grid” like that of achondroplasia can give an additional view since it gives an idea of the degree of shortness as well as a visual background for following tempo of growth. This might be valuable in extreme short stature conditions like acromesomelic dysplasia type Maroteaux, pseudoachondroplasia and metatrophic dysplasia just to name a few. We thus encourage to use the achondroplasia charts when following growth in skeletal dysplasias with extreme short stature.

## CONFLICT OF INTEREST

Lars Hagenäs has received a research grant and consultant fee from BioMarin Europe Ltd. and has received consultant fee from Ascendis Inc.

## AUTHOR CONTRIBUTIONS

Luitgard Neumeyer, Andrea Merker, and Lars Hagenäs conceived of the presented idea. Luitgard Neumeyer conducted all auxological measurements and drafted the scope of the growth charts. Andrea Merker coordinated most of the technical details, performed all calculations and designed the illustrations. Lars Hagenäs compiled all clinical aspects and drafted the article. All authors discussed the results and contributed to the final article.

## Supporting information


**Appendix** **S1**: Achondroplasia growth chart booklet boys

**Appendix** **S2**: Achondroplasia growth chart booklet girls

**Appendix** **S3–S6**: Complementary growth charts to described examples

**Appendix** **S7**: Recommendations for measuring technique and important areas of clinical surveillance to be documented in the chart booklet

## Data Availability

Data sharing is not applicable to this article as no new data were created or analyzed in this study.
