# Supplementary material for: Clinical charts for surveillance of growth and body proportion development in achondroplasia and examples of their use
Source: Am J Med Genet A. 2020 Nov 21;185(2):401–12. doi: 10.1002/ajmg.a.61974 (PMC7839678; doi:10.1002/ajmg.a.61974)
Supplement: Supplementary file 1 — Appendix S1: Achondroplasia growth chart booklet boys [file AJMG-185-401-s001.pdf]

Date of birth	Surname	First name	**Male** ♂

Family's contact details

....

State of health of the child

Problems during pregnancy ☐ no ☐ yes, ... ☐ my child is adopted

Problems at birth ☐ no ☐ yes, ... delivery (weeks/ days): .... ☐ sectio

Birth details length (cm): .... weight (g): .... head circumference (cm): ....

I evaluate my child's motor development as ☐ normal ☐ late ☐ very late.

I evaluate my child's speech development as ☐ normal ☐ early ☐ late.

ACHONDROPLASIA GROWTH CHARTS

Details on growth and state of health in the family

Allergies, illnesses or conditions in the mother's or father's family:

....

....

....

Parents	Current height (cm)/ weight (kg)	Pubertal development	Comments
Mother		.... Menarche age (years)	
Father		☐ normal<br>☐ early<br>☐ late	

Siblings	brother/ sister	brother/ sister	brother/ sister	brother/ sister
Age (yrs, mo)				
Height (cm)				

Achondroplasia Guidelines for Health Supervision adapted from Trotter et al., 2005*

Evaluation	Recommended ages	Date performed							
Neurology									
Physiotherapy									
Orthopaedics	yearly after walking age								
Audiology/ ENT	at 12mo, 24mo then yearly								
Orthodontics	yearly as of 4y								
Radiology of legs									
Review of sleeping position/ snoring/ apnea									
Respiratory/ sleep study									
Gait assessment									
Support groups Families network									
Adjustment of home/ school environment (occupational therapist)									

*Trotter et al. American Academy of Pediatrics Committee on Genetics. Health supervision for children with achondroplasia. Pediatrics. 2005; 116:771-83.

Date of birth	Surname	First name	**Male** ♂

Length/height, weight and head circumference for age, 0-48 months, Achondroplasia

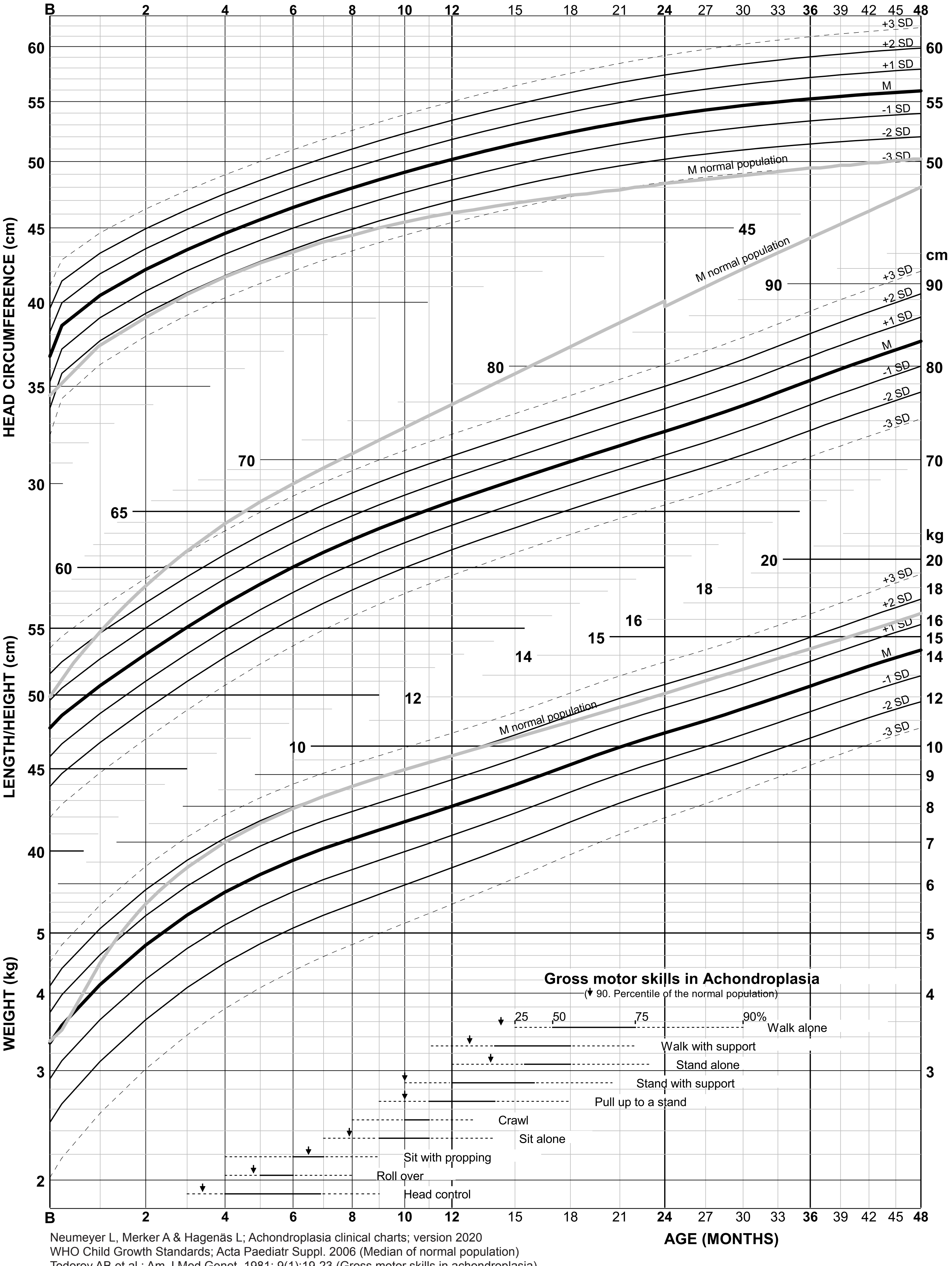

Neumeyer L, Merker A & Hagenäs L; Achondroplasia clinical charts; version 2020
WHO Child Growth Standards; Acta Paediatr Suppl. 2006 (Median of normal population)
Todorov AB et al.; Am J Med Genet. 1981; 9(1):19-23 (Gross motor skills in achondroplasia)

Date of birth	Surname	First name	Male ♂

Height and weight for age, 4-20 years, Achondroplasia

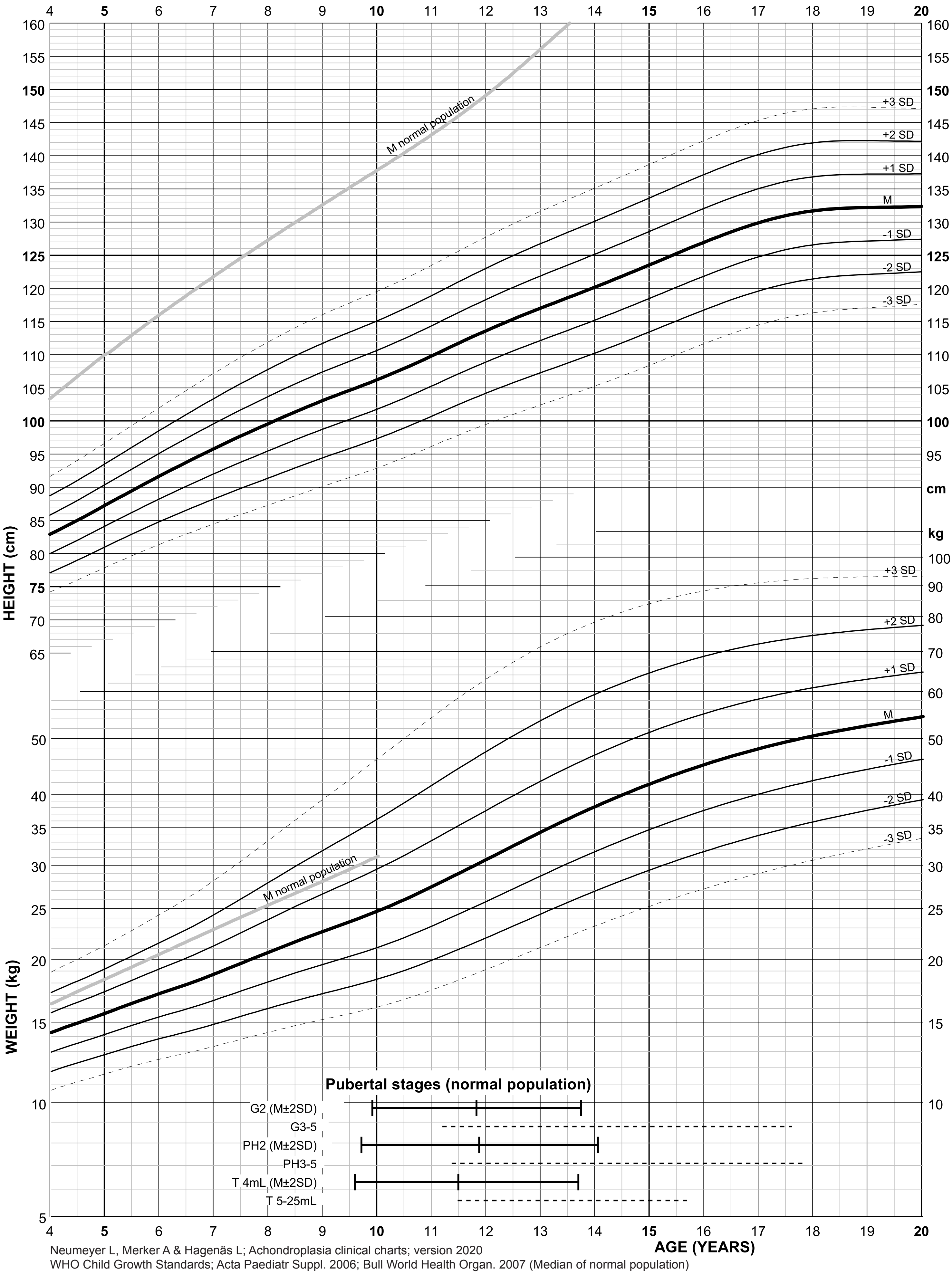

Neumeyer L, Merker A & Hagenäs L; Achondroplasia clinical charts; version 2020
WHO Child Growth Standards; Acta Paediatr Suppl. 2006; Bull World Health Organ. 2007 (Median of normal population)
Juul A et al.; Int J Androl. 2006; 29(1):247-55 (Pubertal stages of normal population)

Date of birth	Surname	First name	Male ♂

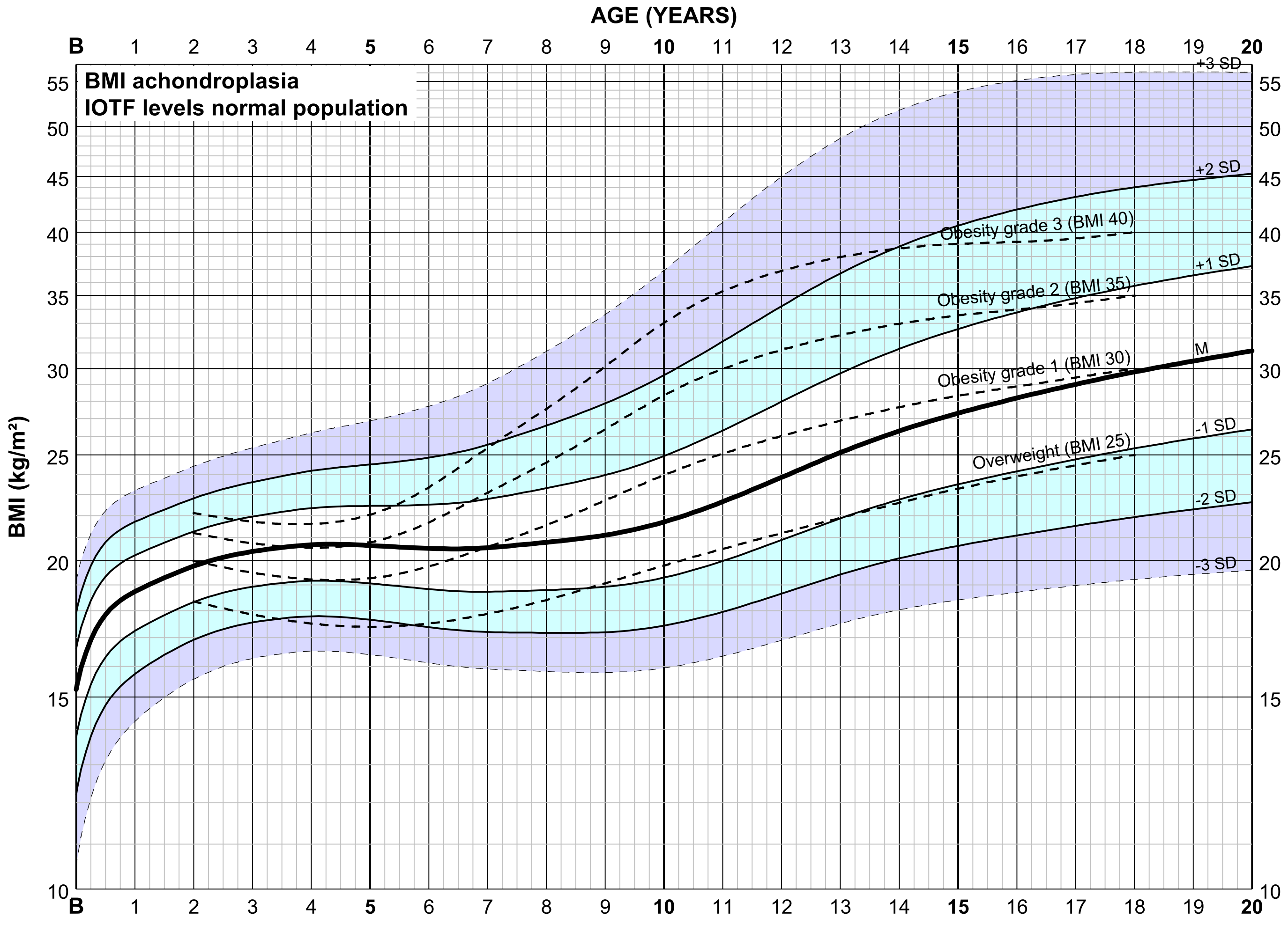

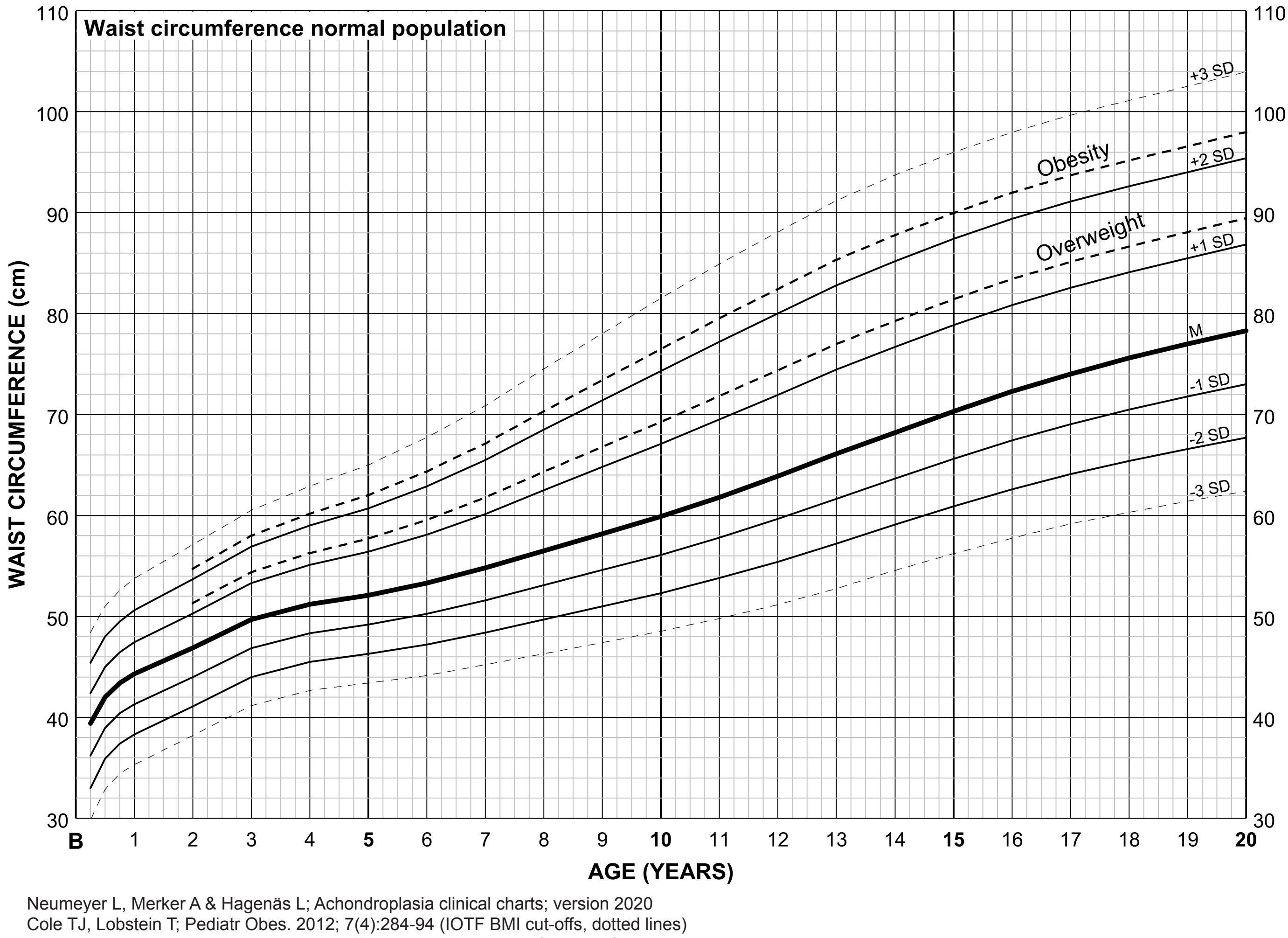

Neumeyer L, Merker A & Hagenäs L; Achondroplasia clinical charts; version 2020
Cole TJ, Lobstein T; Pediatr Obes. 2012; 7(4):284-94 (IOTF BMI cut-offs, dotted lines)
Fredriks M et al.; Eur J Pediatr. 2005; 164(4):216-22 (Waist circumference of normal population)

Date of birth	Surname	First name	Male ♂

Sitting height and sitting height / height ratio for age, 2 to 20 years, Achondroplasia

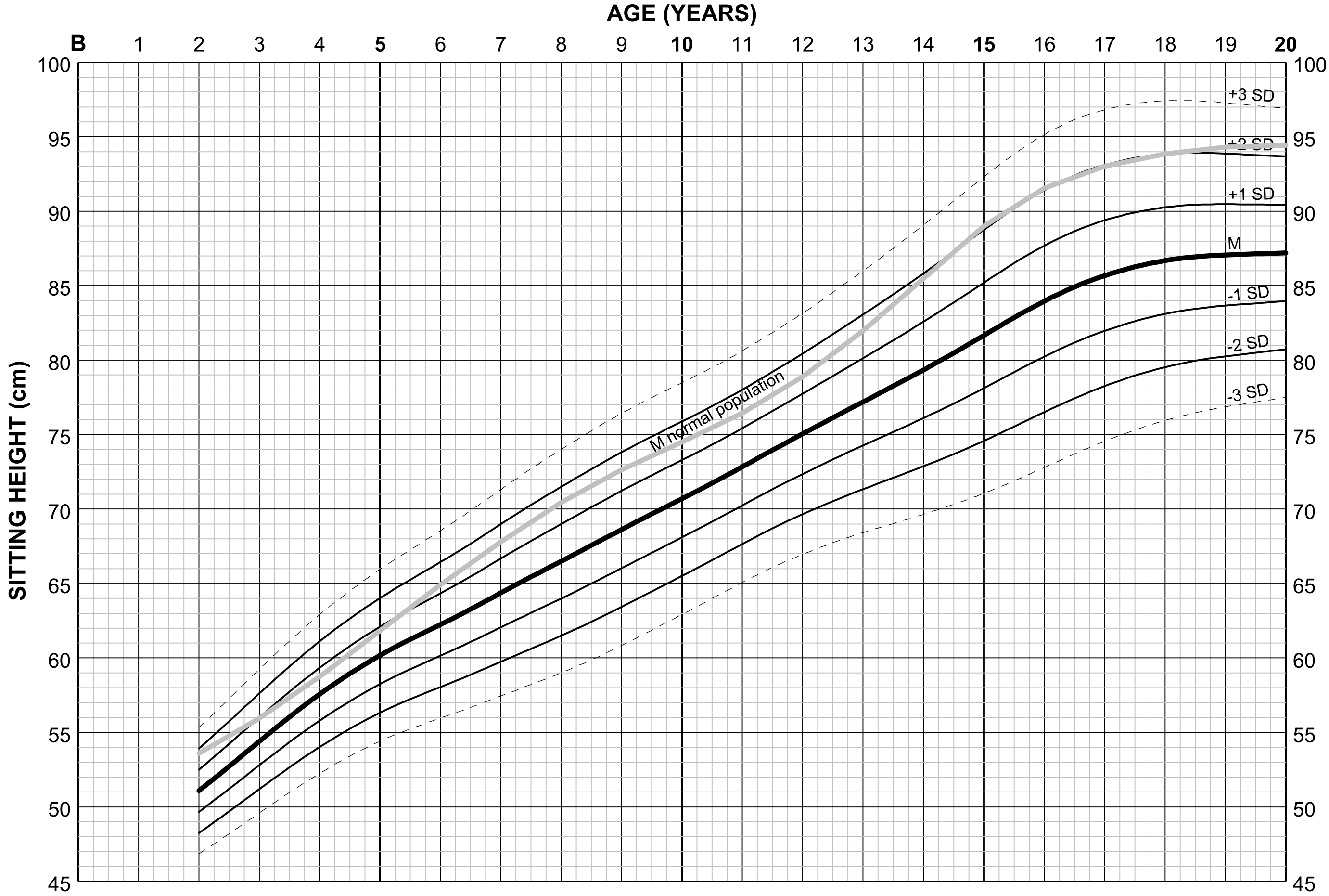

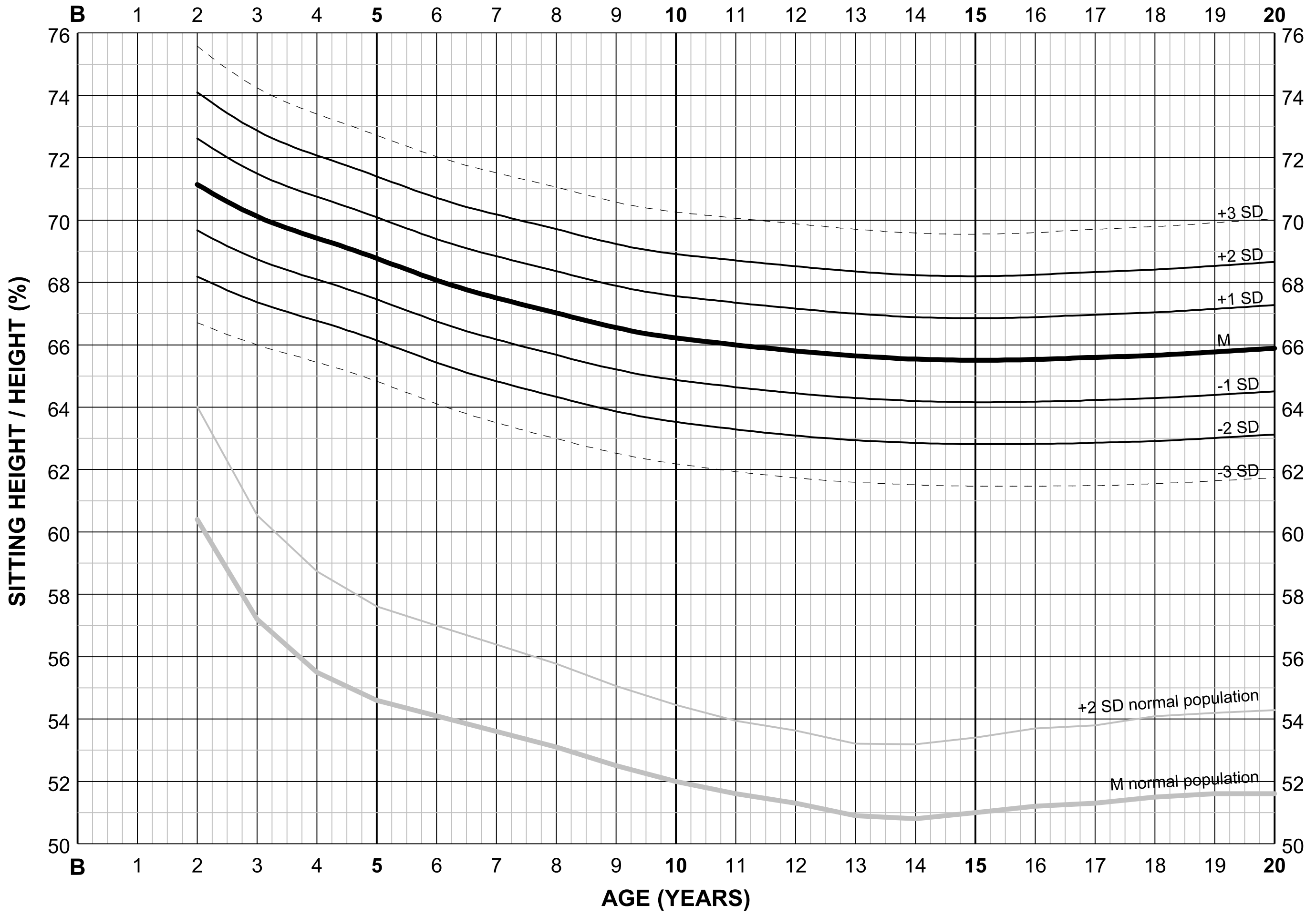

Neumeyer L, Merker A & Hagenäs L; Achondroplasia clinical charts; version 2020
Fredriks M et al.; Arch Dis Child. 2005; 90(8):807-12 (Median of normal population)

Date of birth	Surname	First name	Male ♂

Arm span and leg length (SILL) for age, 2 to 20 years, Achondroplasia

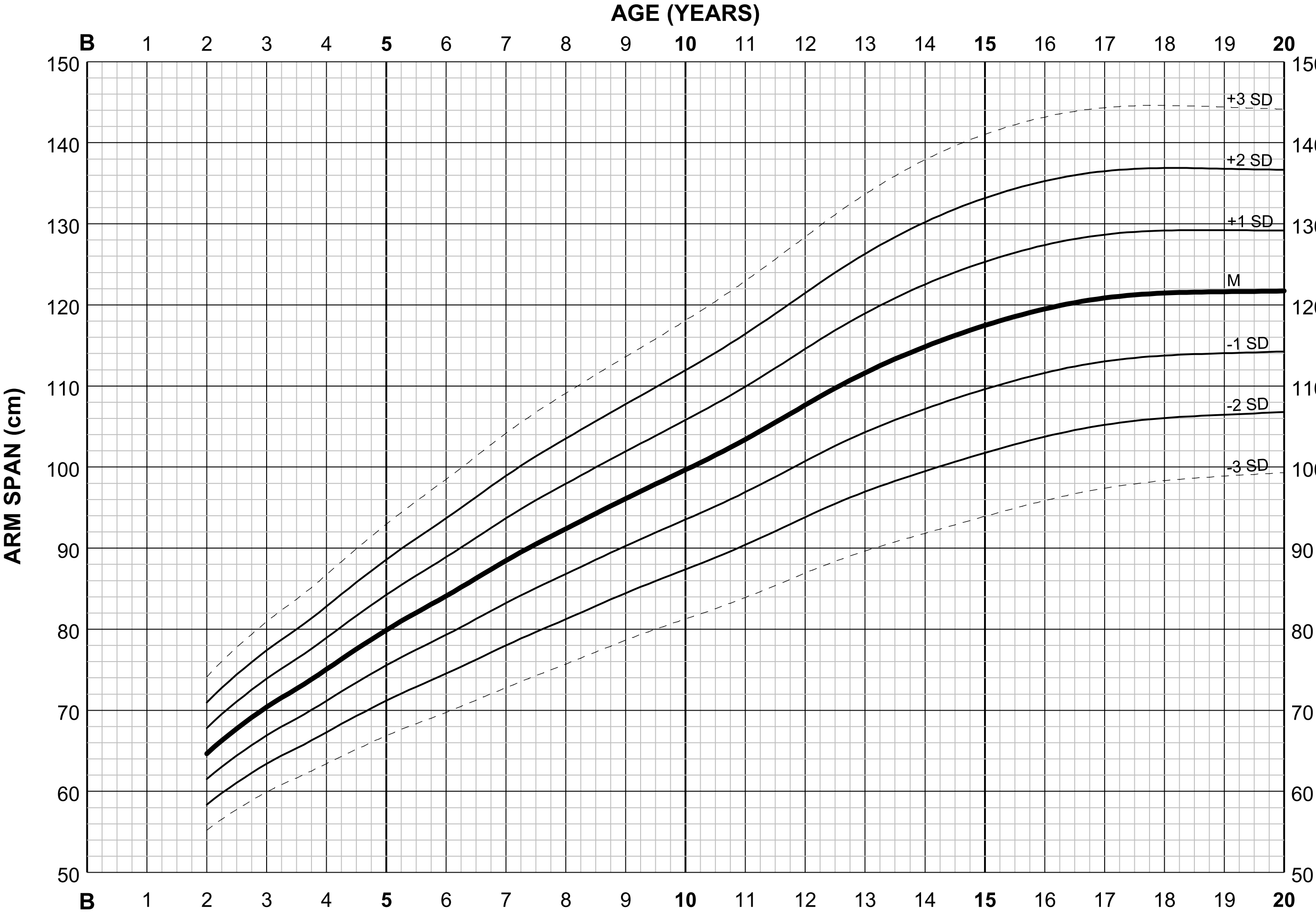

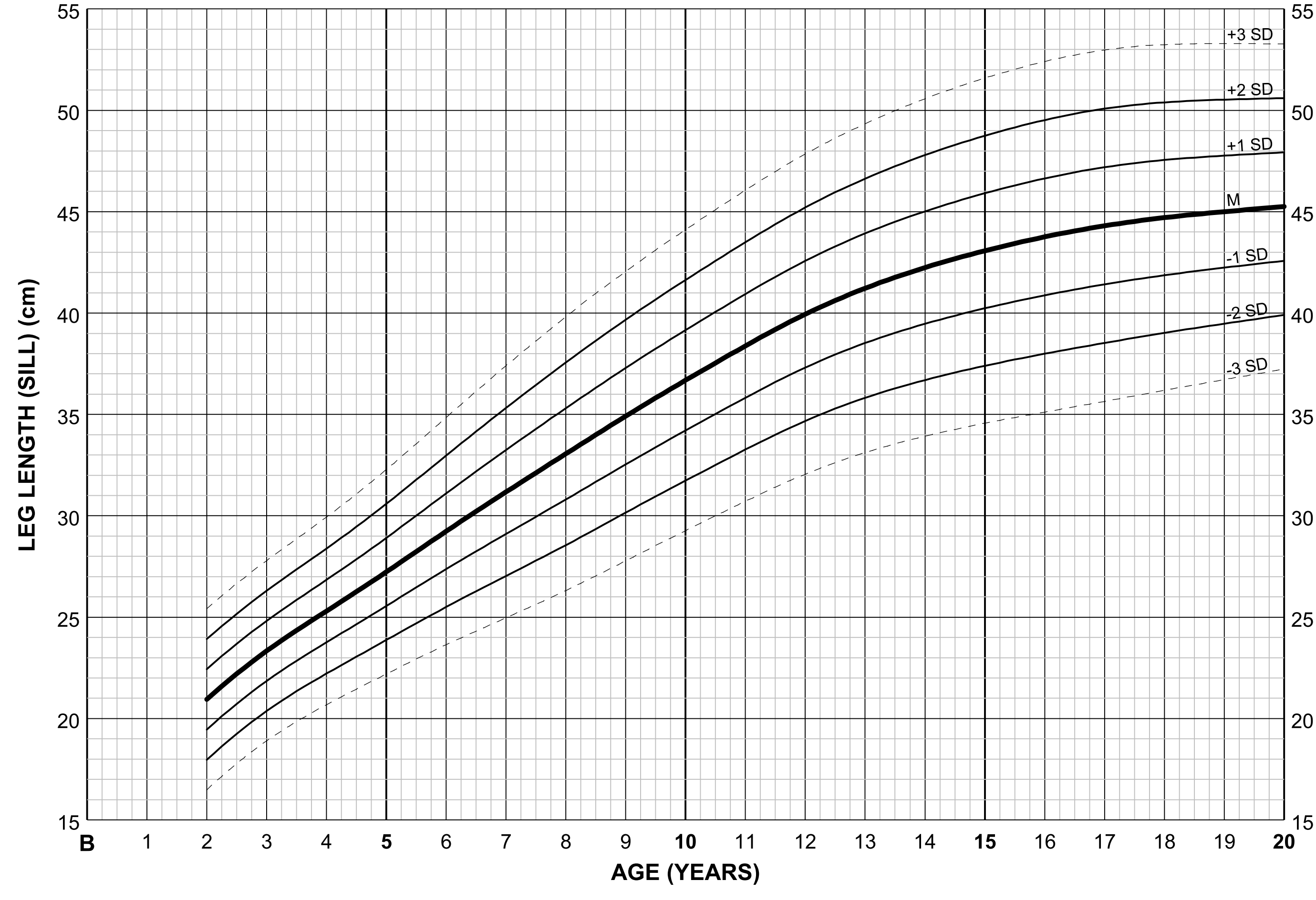

Neumeyer L, Merker A & Hagenäs L; Achondroplasia clinical charts; version 2020

Date of birth	Surname	First name	Male ♂

Surveillance of head circumference and neurological function, Achondroplasia

	Date(s) performed
MRI brain, spine	
Ultrasound ventricles	
Radiology/ CT spine	
Craniocervical decompression/ shunt	
Symptomes spinal stenosis, Thigh angle (L/R) (in Thomas position)*	

* Siebens AA, et al. Curves of the achondroplastic spine: a new hypothesis. Johns Hopkins Med J. 1978; 142:205-10.

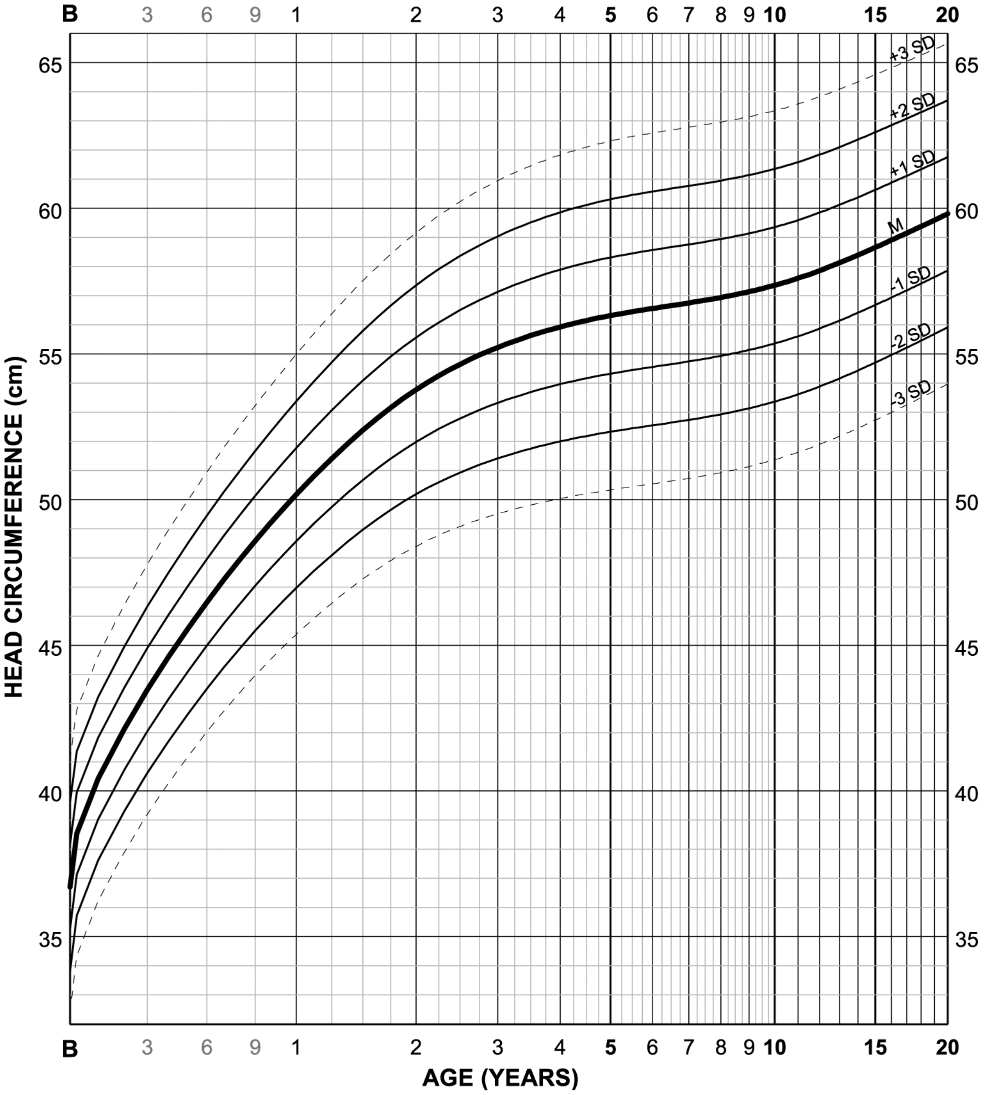

Foot length for age, 0 to 20 years, Achondroplasia

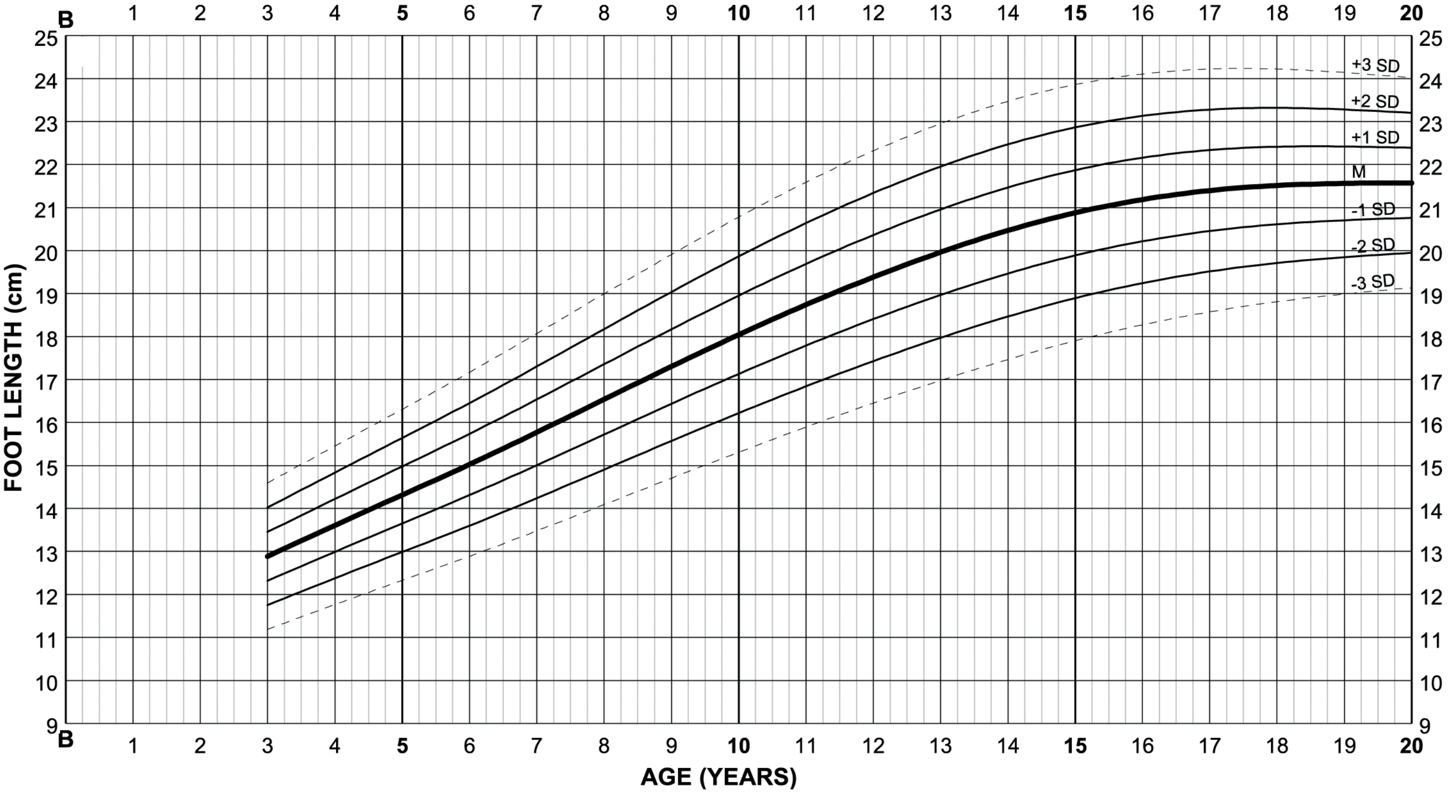

Neumeyer L, Merker A & Hagenäs L; Achondroplasia clinical charts; version 2020

PC PAL - GrowthXP

Date of birth	Surname	First name	**Male** ♂

Measurements

Please indicate details on pubertal development directly in the puberty box in the growth chart.

	Height	Weight/ BMI	Sitting height (cm/ %)	Arm span	Head circumference	
Mother						
Father						

**Date** / **Age (yr, mo)**	**Length/ height (cm)**	**Weight (kg)** / **BMI (kg/m²)**	**Sitting height (cm)** / **SH/H %**	**Arm span (cm)**	**Head circumference (cm)**	**Comment** (e.g. orthopaedics, other measurements, difficulties when measuring, e.g. extension defects)
