# Supplementary material for: Clinical charts for surveillance of growth and body proportion development in achondroplasia and examples of their use
Source: Am J Med Genet A. 2020 Nov 21;185(2):401–12. doi: 10.1002/ajmg.a.61974 (PMC7839678; doi:10.1002/ajmg.a.61974)
Supplement: Supplementary file 3 — Appendix S3–S6: Complementary growth charts to described examples [file AJMG-185-401-s003.pdf]

Supporting information S3-S6

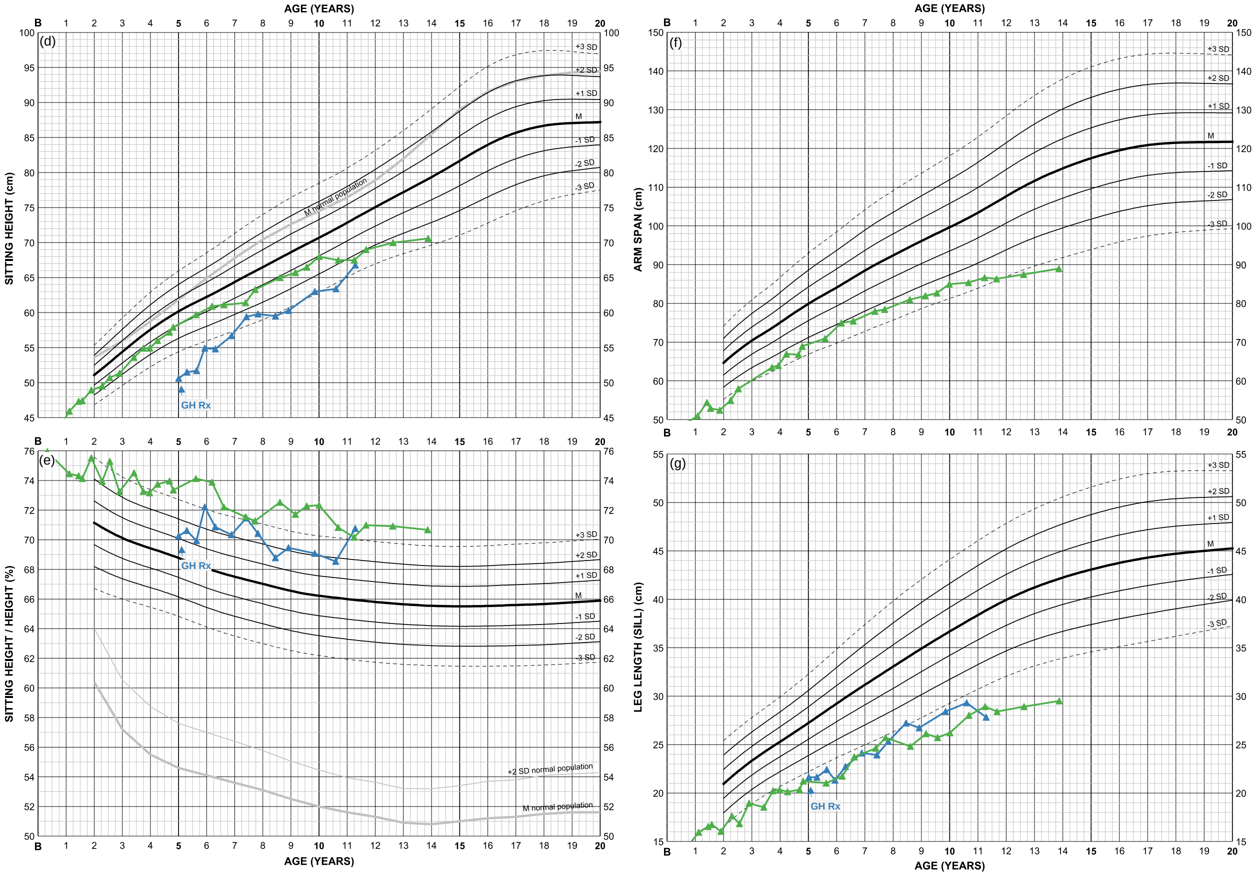

Supporting information S3 - Body proportion charts of the boys described in Figure 2: Leg length develops abnormal in both boys. In one boy (in blue) also sitting height develops outside normal ranges contributing to a for achondroplasia atypical height position. Height of the other boy (in green) is affected mainly due to the short legs. Relative sitting height becomes therefore further distorted in the body proportion chart. Arm span seems affected to a lesser degree. Normal innervation and mobility of an extremity seems important also in achondroplasia where leg growth is already maximally growth restricted.

Supporting information S3-S6

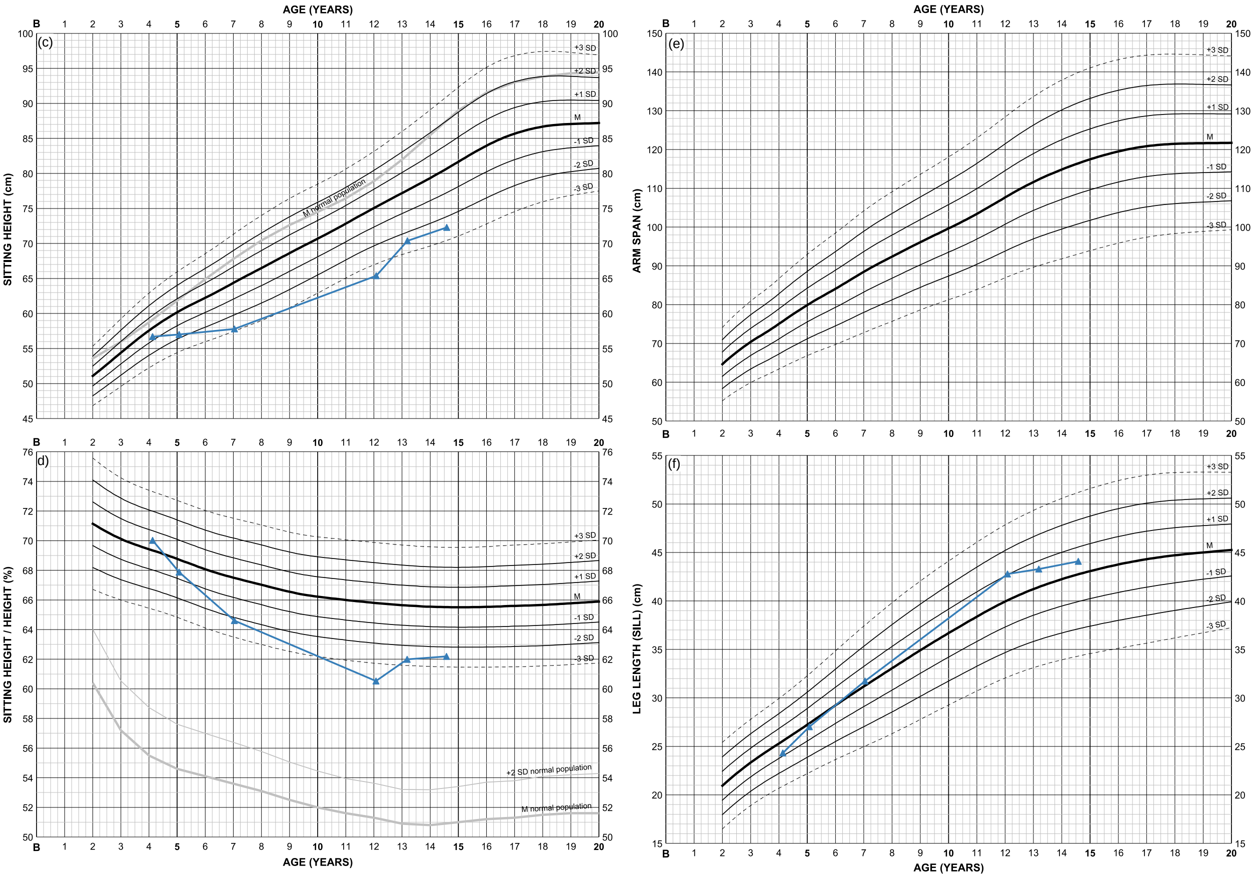

Supporting information S4 - Body proportion charts of a boy with achondroplasia and Mb Down described in Figure 3: (Absolute) sitting height develops in the lower achondroplasia range during childhood, compensated by less affected growth of the legs. His leg length follows achondroplasia mean. Relative sitting height and body disproportion is therefore less pronounced. Arm span data is not available.

Supporting information S3-S6

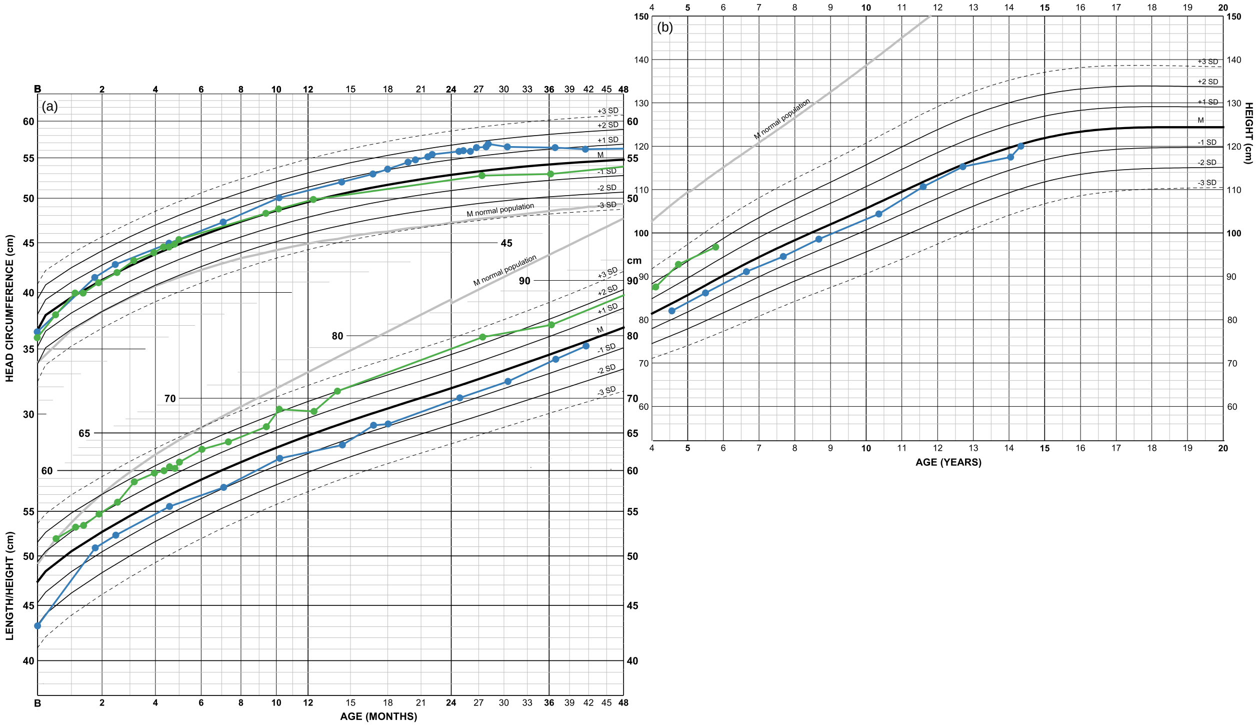

Supporting information S5 – Growth charts of the girls with different FGFR3 mutations described in Figure 7: one with Gly380Arg/ achondroplasia (in blue, also shown in **Figure 5**) and one with Asn540Lys/ hypochondroplasia (in green). Sitting height (shown in **Figure 7**) is similar yet a less affected leg growth contributes to a better height position as well as less severe body disproportions in the girl with the common hypochondroplasia mutation.

Supporting information S3-S6

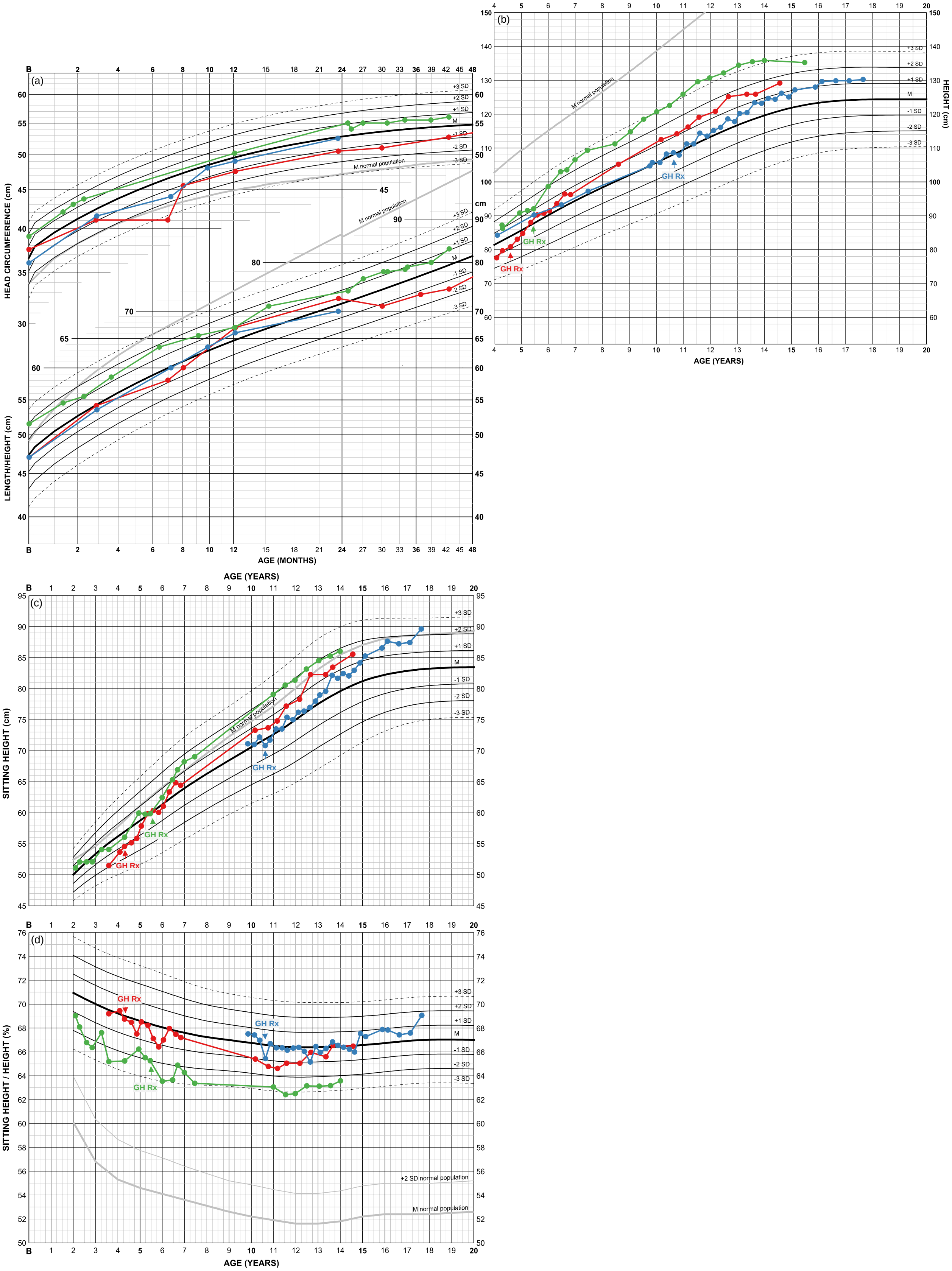

Supporting information S3-S6

Supporting information S6 - Growth and sitting height charts of the girls described in Figure 8: all are treated with growth hormone (GH Rx) from indicated ages. Note that these cases are selected only to show the usability of the charts and cannot be regarded as representative for GH Rx in achondroplasia.

The girl in green has a high height position from early ages. After a clear treatment response in height, a change of +1.4 SDS during the first treatment year, her adult position (+2.4 SDS) grossly reiterates that of early childhood. Relative sitting height develops at a favorable position.

The girl in red starts GH Rx already at 4.6 years at a height position of -0.9 SDS. The treatment causes a distinct acceleration in height, which is sustained at least until 14.6 years when height is 129 cm (+1.6 SDS). Relative sitting height position maintains at or just below the mean suggesting that growth stimulation is parallel for trunk and extremities.

Height development of the girl in blue seems initially not to be influenced by GH Rx, with a position changing from +0.1 at 10.6 years to +0.5 SDS at 11.6 years of age. A sustained height acceleration is present first after 13 years attaining 130.1 cm (+1.2 SDS). Relative sitting height shows a trend towards increasing disproportion due to a late preference for trunk growth.
