# Supplementary material for: Clinical charts for surveillance of growth and body proportion development in achondroplasia and examples of their use
Source: Am J Med Genet A. 2020 Nov 21;185(2):401–12. doi: 10.1002/ajmg.a.61974 (PMC7839678; doi:10.1002/ajmg.a.61974)
Supplement: Supplementary file 4 — Appendix S7: Recommendations for measuring technique and important areas of clinical surveillance to be documented in the chart booklet [file AJMG-185-401-s004.pdf]

Supporting information S7

Recommendations for measuring technique in achondroplasia: It is important that reproducible measuring technique is used taking into account that cooperation with the child is a prerequisite to obtain reliable results. Blinded measurements are recommended; i.e. reading the value only when the desired position is obtained rather than trying to approach a previous measure at the same measuring occasion. We therefore share here experiences in measuring technique for growth and body proportion variables in relation to achondroplasia.

Length and height: Measuring supine length before the child can stand demands two persons to secure that both heels contact the bottom plate of the measuring board to get reproducible results. In standing position, when a significant lordosis is present, external pressure could be used to align the back towards the measuring support area as much as possible. If using stretching technique in standing, all subsequent measurements should be stretched values. Height may, as for normal children, decrease significantly between morning and afternoon due to the normal compression of disks during the day. When following an individual's growth closely, the time of day for measurement is thus an issue and is preferentially standardized.

Head size: Head size is measured as maximum circumference using a non-stretchable not to narrow measuring tape. Head circumference is only included in the infant growth chart page since the majority of head size, i.e. more than 90 percent, is achieved at four years of age.

Weight, BMI and waist circumference: Weight is measured with the child being undressed or in light clothing using a calibrated (digital) scale. BMI, as retrieved from weight and height, might be more sensitive to measuring inaccuracies and can therefore appear irregular when plotted. Absolute BMI level of an individual might be less interesting but its development can give valuable information about a child's ability to accumulate fat. Waist circumference is measured mid-way between last rib and iliac crest after normal exhalation. Location of "correct" landmark is a repeated challenge even for those with long measuring experience.

Sitting height: Also measuring sitting height demands reproducible measuring technique and smooth cooperation with the child. A more or less redressable, i.e. non-fixed, curvature of the spine will increase individual variability between measurements. As percentage of total height, i.e. "relative sitting height", this ratio is sensitive to measuring inaccuracies and individual development may therefore appear somewhat uneven, similar to BMI values. Expressing relative sitting height position in SDS of normal population can take on absurdly high levels due to rapidly thinning SD channels in the normal population reference (Fredriks et al., 2005).

Arm span and (subischial) leg length: Arm span should be measured as the longest dorsal measure between fingertips of horizontally stretched arms with the back against the wall. If external stretching (by two investigators) is applied (recommended) this technique should be used also at subsequent measuring occasions. Pencil markings can be done before the distance between these is measured with a tape or a measuring stick. Varying degree of flexion contracture of elbows, usually not accessible for stretching, may influence the measure. Similarly, a positional varus of the knee in standing will influence the height measurements and thus also calculation of leg length. Leg length is calculated as difference between length/ height and crown-rump length/ sitting height.

Supporting information S7

Other important areas of clinical surveillance to be documented in the chart booklet: The following areas, adapted from guidelines for health supervision in achondroplasia (Pauli, 2019; Trotter & Hall, 2005; Wright & Irving, 2012), are worthwhile to be recorded in the chart compilation:

1) Investigation by MRI technique of medullar integrity in craniocervical area, preferably also covering the whole spinal canal. Narrowness of foramen magnum and spinal canal is an almost constant feature in achondroplasia, contributing to neurological and respiratory morbidity. Important to note is that association between influence on medullary integrity like impression at foramen magnum level, edema or gliosis or even syringe development and clinical neurological or respiratory symptoms might be unreliable (Cheung et al., 2020; Sanders, Sheldon, & Charrow, 2018). Therefore neuro-radiological investigation should as a routine be done already in neonatal period (Trotter & Hall, 2005). Normally a liquor space should circumvent the medulla at foramen magnum level.

2) Investigation of respiratory function at regular intervals at all ages with regard to snoring, abnormal head position during sleep, apnoic periods; carbon dioxide retention, increased blood pressure, hypoxia and evaluation of need for nighttime respiratory support. In achondroplasia dimensions of throat and upper airways are often reduced causing breathing difficulties, at times further complicated by central apnea tendency (Tenconi et al., 2017; Zaffanello et al., 2017).

3) Regular assessment of ENT status with regard to disproportionally increased size of tonsils and adenoid, oromotor function as well as checking for otitis media and possible hearing loss. These items are often causing problems in achondroplasia (Afsharpaiman, Saburi, & Waters, 2013; Sisk, Heatley, Borowski, Leverson, & Pauli, 1999).

4) Orthopedic dynamics and problems. Examination of postural position and walking. The often distinct thoracolumbar kyphosis during infancy should resolve after walking starts. Laxity of knee joints may cause positional varus of legs in standing and walking, sometimes exaggerated by varus deformity of tibia. Symptoms of spinal stenosis with tiredness and pain in lumbar area after walking and/ or paresthesia's of legs may present already in childhood. Sometimes symptoms are accompanied by secondary enuresis. Development of significant lordosis that is exaggerated in standing and walking further reduces spinal canal dimensions and provoke stenosis symptoms. An informative discussion on the influence of postural position on the space in spinal canal for the medulla and its outgoing nerves is found in Siebens et al (Siebens, Hungerford, & Kirby, 1978).

5) Preventing truncal obesity. Since limited physical activity may be present, caused by short legs together with abnormal position and limited range of movements of hip joints and waggling gait due to positional varus of knee joints, development of truncal obesity may be seen as a disease specific handicap.

Supporting information S7

References:

Afsharpaiman, S., Saburi, A., & Waters, K. A. (2013). Respiratory difficulties and breathing disorders in achondroplasia. *Paediatric Respiratory Reviews, 14*(4), 250–255. http://doi.org/10.1016/j.prrv.2013.02.009

Cheung, M. S., Irving, M., Cocca, A., Santos, R., Shaunak, M., Dougherty, H., … Thompson, D. (2020). Achondroplasia Foramen Magnum Score: screening infants for stenosis. *Archives of Disease in Childhood*. http://doi.org/10.1136/archdischild-2020-319625

Fredriks, M. A., van Buuren, S., van Heel, W. J. M., Dijkman-Neerincx, R. H. M., Verloove-Vanhorick, S. P., & Wit, J. M. (2005). Nationwide age references for sitting height, leg length, and sitting height/height ratio, and their diagnostic value for disproportionate growth disorders. *Archives of Disease in Childhood, 90*(8), 807–812. http://doi.org/10.1136/adc.2004.050799

Pauli, R. M. (2019). Achondroplasia: a comprehensive clinical review. *Orphanet Journal of Rare Diseases*, *14*(1), 1. http://doi.org/10.1186/s13023-018-0972-6

Sanders, V. R., Sheldon, S. H., & Charrow, J. (2018). Cervical spinal cord compression in infants with achondroplasia: should neuroimaging be routine? *Genetics in Medicine : Official Journal of the American College of Medical Genetics*. http://doi.org/10.1038/s41436-018-0070-0

Siebens, A. A., Hungerford, D. S., & Kirby, N. A. (1978). Curves of the achondroplastic spine: a new hypothesis. *The Johns Hopkins Medical Journal*, *142*(6), 205–210.

Sisk, E. A., Heatley, D. G., Borowski, B. J., Leverson, G. E., & Pauli, R. M. (1999). Obstructive sleep apnea in children with achondroplasia: surgical and anesthetic considerations. *Otolaryngology--Head and Neck Surgery : Official Journal of American Academy of Otolaryngology-Head and Neck Surgery*, *120*(2), 248–254. http://doi.org/10.1016/S0194-5998(99)70414-6

Tenconi, R., Khirani, S., Amaddeo, A., Michot, C., Baujat, G., Couloigner, V., … Fauroux, B. (2017). Sleep-disordered breathing and its management in children with achondroplasia. *American Journal of Medical Genetics. Part A, 173*(4), 868–878. http://doi.org/10.1002/ajmg.a.38130

Trotter, T. L., & Hall, J. G. (2005). Health supervision for children with achondroplasia. *Pediatrics*, *116*(3), 771–783. http://doi.org/10.1542/peds.2005-1440

Wright, M. J., & Irving, M. D. (2012). Clinical management of achondroplasia. *Archives of Disease in Childhood*, *97*(2), 129–134. http://doi.org/10.1136/adc.2010.189092

Zaffanello, M., Cantalupo, G., Piacentini, G., Gasperi, E., Nosetti, L., Cavarzere, P., … Antoniazzi, F. (2017). Sleep disordered breathing in children with achondroplasia. *World Journal of Pediatrics : WJP*, *13*(1), 8–14. http://doi.org/10.1007/s12519-016-0051-9
